# Heart Rate in Hypertension: Review and Expert Opinion

**DOI:** 10.1155/2019/2087064

**Published:** 2019-02-19

**Authors:** Jamshed Dalal, Arup Dasbiswas, Immaneni Sathyamurthy, Srinivasa Rao Maddury, Prafulla Kerkar, Sandeep Bansal, Joy Thomas, Sankar Chandra Mandal, Soura Mookerjee, Sivakadaksham Natarajan, Viveka Kumar, Nishith Chandra, Aziz Khan, R. Vijayakumar, J. P. S. Sawhney

**Affiliations:** ^1^Centre for Cardiac Sciences, Kokilaben Dhirubhai Ambani Hospital, Mumbai, India; ^2^Department of Cardiology, NRS Medical College, Kolkata, West Bengal, India; ^3^Dept. of Cardiology, Apollo Hospitals, Chennai, India; ^4^Department of Cardiology, Care Hospitals, Hyderabad, India; ^5^Department of Cardiology, KEM Hospital, Mumbai, Maharashtra, India; ^6^Department of Cardiology, Safdarjung Medical College, Delhi, India; ^7^Dr. Joy Thomas Heart Care, Bharathi Salai, Mogappair West, Chennai, India; ^8^Belle Vue Clinic, Brahmachari Street, Kolkata, India; ^9^Welkin Medicare, Garia, Kolkata, India; ^10^Siva's Cardio Diabetic Care, Royapettah, Chennai, India; ^11^Max Hospital, Saket, New Delhi, India; ^12^Fortis Escorts Hospital, New Delhi, India; ^13^Crescent Hospital & Heart Centre, Dhantoli, Nagpur, Maharashtra, India; ^14^Billroth Hospital, Mandaveli, Chennai, India; ^15^Dept. of Cardiology, Sir Ganga Ram Hospital, New Delhi, India

## Abstract

Heart rate (HR) is strongly associated with both peripheral and central blood pressures. This association has implications in hypertension (HTN) prognosis and management. Elevated HR in HTN further elevates the risk of adverse outcomes. Evidence suggests that HR is an independent risk factor for cardiovascular (CV) and total mortality in patients with HTN. With objective to engage physicians and researchers in India to identify and discuss the implications related to HR management in HTN, experts in the HTN management provided consensus recommendations. The key expert recommendations included the following. (i) Heart rate (HR) has inverse relationship with the central aortic pressure, whereby reduction in HR is associated with an increase in central aortic pressure. This counter-balances the benefit of HR reduction with the harmful effects of rising central aortic pressure. (ii) Increase in the resting HR is associated with increased risk of incident HTN. A linear association between the two is observed especially in individuals with HR >80 bpm. (iii) A reduced HR variability further adds to the propensity for the development of HTN, especially in men. (iv) Each 10 beats per minute increase in the resting HR can substantially increase the risk of adverse CV and mortality outcomes. On treatment HR provides a better prognostic guide. (v) Ambulatory HR with day-time and night-time HR evaluation may also suggest different impact on outcomes. (vi) Target HR in patients with HTN remains unclear. Generally, HR<70 bpm on beta blocker (BB) treatment is advised which may be further lowered in patients with comorbidities like heart failure and coronary artery disease. (vii) Adopting healthy lifestyle approaches to keep check on BP and HR is essential. (viii) Use selective beta-1 blocker in* symptomatic* cases with elevated HR beyond 80-85 mmHg. BBs are expected to benefit by lowering HR by nearly 10 bpm. Preference should be given to newer beta-blockers which reduce HR and both peripheral and central blood pressure to derive comprehensive advantage of this dual action. (ix) It still remains unclear whether reducing HR in HTN without comorbidities alters the CV and mortality outcomes.

## 1. Introduction

Hypertension (HTN) is a widely prevalent major cardiovascular (CV) risk factor [1]. The risk factors for development of HTN include age, smoking, alcohol, obesity, diabetes, renal damage, and others [2]. The other risk factors that might contribute to pathogenesis of HTN are often neglected and one such risk factor is heart rate (HR). Increase in HR is associated with increase in blood pressure (BP). A 3-4 times higher risk of HTN has been reported with increase in HR even after controlling the traditional risk factors [3]. In young hypertensives, baseline HR and changes in HR in first few months are found to be associated with sustained HTN [4]. Additionally, increasing HR in patients with HTN is associated with adverse CV outcomes [5]. Thus, management of raised HR in HTN is crucial. At present, European Society of Hypertension (ESH) provides some recommendations for HR measurement in HTN, approach to management of elevated HR and carrying out supplementary research to provide solutions for problematic areas [6]. In Indian context where prevalence of HTN is colossal, we felt that there is need to enlighten the importance of HR in HTN to the physicians, advise them on issues with management of HR in HTN, and identify the critical areas to carry out further research in field of HR in HTN. This document highlights important literature along with expert opinion from authors for management of elevated HR in patients with HTN. The discussion of the consensus was limited to heart rate in sinus rhythm and does not include the abnormal heart rate conditions like tachy- or brady-arrhythmias.

## 2. HR and Relation to Peripheral and Central Blood Pressure

Changes in both peripheral and central pressures are strongly associated with HR and this relationship is quite complex. The difference between central pressure and peripheral blood pressure can be up to 20 mmHg [7]. With regard to CV outcomes, the strong heart study demonstrated that the central pulse pressure is more strongly associated with vascular hypertrophy, extent of atherosclerosis, and CV events than peripheral pulse pressure [8]. Increase in HR is known to increase the peripheral pressure whereas it reduces the central pressure. These antagonistic effects of the HR on the two pressures raise concern with respect to selection of the antihypertensive medications [7]. Among various antihypertensives, beta-blockers (BBs) reduce the HR besides reducing BP. The CAFÉ study observed significant reduction in the central aortic pressure with amlodipine-based but not with the atenolol-based treatment despite similar reduction in the brachial pressures. Therefore, differences in central pressure with two treatments could possibly explain the differences in outcomes reported in the study [9]. A further analysis from this CAFÉ data observed that HR has no effect on brachial pressure, but a significant inverse relationship exists between HR and central pressure. Multivariate analysis showed that HR was the major determinant of the central pressure. Thus, atenolol-based treatment was associated with less effective central pressure reduction relative to decrease in the peripheral pressure [10]. This inverse association with the central aortic pressure has been found to be associated with increased risk of CV events with the use of BB in HTN. A metanalysis of randomized controlled trials assessing HR changes with the use of BB in HTN reported increased risk of all-cause deaths (r = -0.51; p < 0.0001), CV deaths (r = -0.61; p < 0.0001), myocardial infarction (r = -0.85; p < 0.0001), stroke (r = -0.20; p = 0.06), or heart failure (r = -0.64; p < 0.0001) with lower HR in patients of HTN [11]. This implies important therapeutic considerations for selecting antihypertensive drugs affecting HR. However, some authors have advised caution in interpretation of the results of this metanalysis [12].

*Expert Opinion*. HR has inverse relation with the central aortic pressure which affects CV outcomes in patients with HTN. This has implications in selecting antihypertensive drugs that affect HR. A preference to the drugs like newer BB which reduce HR as well as peripheral and central pressure is advised.

## 3. Resting HR and Risk of Incident HTN

In lieu of these effects, the question arises whether increase in HR increases the risk of incident HTN. Studies done in different regions worldwide suggested that increase in HR from baseline is associated with increased risk of HTN and these are discussed briefly below.

A cross-sectional survey from India—The BEAT survey—performed in 3743 young (18 to 55 years) hypertensive reported average resting heart rate of 82.79±10.41 bpm and BP of 146.82±15.46 / 89.08±8.8 mmHg. HR had significant positive correlation with both SBP (r = 0.247, p<0.01) and DBP (r =0.219, p<0.01). The resting HR was elevated in Indian population as observed in this survey which can impact CV morbidity and mortality [13].

In the Kailuan cohort study, Wang et al. from China studied 31507 participants with mean age of 46.3±11.5 years having no known HTN. During the mean follow-up of 3.5 years, 39.88% developed HTN. In multivariate analysis, significant increase in new onset HTN with increase in the resting HR (p<0.0001) was observed. Further, with increase in the resting HR by 10 bpm, a rise of 8% in HTN was reported [14].

Results of the fifth Korea National Health and Nutrition Examination Survey suggested that men with resting HR of 90 bpm or more were at 2.75-times increased risk of incident HTN, but the same relation was not seen in women. Higher body mass index (>23 kg/m^2^) was also associated with higher risk of incident HTN. Further, a higher HR significantly increased the risk of diabetes and metabolic syndrome in both genders [15]. These proofs clearly suggest a significant increase in incident HTN with increase in resting HR.

Besides increase in HR, variability in HR is also a crucial component. HR variability has also been reported to increase the risk of developing HTN. From the Framingham Heart Study data, patients who were normotensives (BP<140/90 mmHg) and had HR variability (HRV) indices measured at baseline were studied further. Among 1434 patients (633 men and 801 women) identified from the dataset, 244 (119 men and 125 women) developed HTN during the 4-years of follow-up. Adjusted multiple logistic regression analysis suggested association of low-frequency power (LF) with development of HTN in men but not in women. However, authors concluded that a reduced HR variability in normotensive individuals poses greater risk of incident HTN and the dysregulation of autoimmune functions can be observed from early stages of HTN [16].

*Expert Opinion.* An increase in the resting HR is associated with increased risk of incident HTN. A linear association may be observed in individuals with HR >80 bpm. A reduced HR variability may add to the prediction of the development of HTN especially in men.

## 4. HR as a Prognostic Marker

One of the earliest proofs about prognostic importance of HR is from the study of Levy et al., published in JAMA in 1945 [17]. Transient tachycardia was defined as HR (of sinus-origin) being ≥100 bpm and the subsequent HR after a rest or at a later examination being <100 bpm. With increasing age, the frequency of first transient tachycardia was linearly related increasing from 3.7% in patients between 25 and 29 years to 6.6% in patients aged between 50 and 54 years. Transient tachycardia was associated with later development of sustained HTN. Also, presence of transient HTN was associated with increased death rate due to cardiovascular-renal disease beyond 45 years of age. Additionally, presence of transient HTN along with transient tachycardia was associated with twice greater risk of development of sustained HTN and enhanced risk of CV disease related deaths [17]. Evaluation of Framingham study data by Gilman et al. suggested association of HR with mortality in patients of HTN not treated with antihypertensives. Each increase in HR of 40 bpm was associated with nearly two-times higher risk of all-cause mortality and 1.5-times higher risk of CV mortality suggesting HR as independent predictor of mortality in HTN [18].

Evaluation of the data from the VALUE trial suggested incremental risk of cardiac events with rise of HR. In patients with high-risk HTN, every supplemental increment of 10 bpm from baseline was associated with hazard ratio (HR) of 1.16 for composite cardiac outcome. The significant association persisted in individual cardiac events like heart failure (HR 1.24), sudden cardiac death (HR 1.18), MI (HR 1.10), stroke (HR 1.09), and all-cause death (HR 1.19). Among five quintiles of baseline HR, patients in the highest quintile and the second highest quintile had significantly increased risk of the composite and individual cardiac events except stroke. When the highest HR quintile was compared to mean of lower four quintiles, association of the highest quintile with primary composite endpoint, heart failure, and total mortality persisted. Further, an evaluation by controlled and uncontrolled BP reported that the highest HR quintiles had significantly higher rates of composite endpoint in both controlled (+53%, p <0.0001) and uncontrolled (+34%, p<0.002) BP groups. Also, lower four quintiles combined with uncontrolled BP had greater risk of incident composite endpoint (p=0.0035) [19]. Another evaluation of data from LIFE study reported 25% increase in risk of CV deaths and 27% higher risk of all-cause deaths with each increment in HR by 10 bpm. HR of 84 bpm or more was associated with increased risk of CV deaths (89%) and all-cause deaths (97%). This association persisted even in multivariate analysis after adjustment for various factors including treatment with losartan or atenolol [20]. A further analysis suggested that in patients with persistent HR ≥84 bpm or development of HR beyond this threshold was associated with 159% higher risk of heart failure [21]. Similar investigation done in INVEST data showed that baseline resting HR had linear association with adverse outcomes in patients of coronary artery disease (CAD) treated for HTN. Treatment with atenolol-strategy reduced HR more than verapamil-strategy with mean HR of 69.2 vs 72.8 bpm, respectively, by 24 months. However, the incidence of adverse outcomes was similar in two treatments (9.88% vs 9.67%, respectively, p=0.62). Study concluded that on-treatment HR is better predictor of outcomes than resting HR [22]. These analyses highlight the important fact that on-treatment rise in HR is predictive of adverse outcomes and should be considered in all patients under treatment for HTN.

An interesting study by Hozawa et al.—the Ohasama study from Japan—enrolling 1444 individuals from general population without any CV disease (27.4% with HTN) observed that after 12-year follow-up, deaths occurred due to CV disease in 101, non-CV disease in 195, and all-disease mortality seen in 296 participants. When ambulatory HR was assessed, a 10-bpm increment in neither day-time HR nor night-time HR failed to show association with CV deaths. However, both predicted non-CV deaths (HR 1.28 and 1.48, respectively). A 10 bpm rise in night-time HR had independent association with all-cause mortality (HR 1.29) [23]. This highlights the importance of measuring ambulatory HR which can have differential impact on mortality outcomes in general population.

*Expert Opinion*. Resting HR has linear association with adverse outcomes. Each 10 bpm increase in resting HR can substantially increase the risk of adverse CV and mortality outcomes, and on-treatment HR can provide a better prognostic guide. Ambulatory HR with day-time and night-time HR may also have different impact on outcomes.

## 5. Management of Elevated HR in HTN

### 5.1. Target HR in Patients with or without Comorbidities

The evidence from either observational or randomized trials suggesting a specific target at which there can be optimal benefits of HR lowering in patients of HTN is lacking. This restricts identifying specific target HR. For using BB in an Asian population, experts have suggested a target HR of <70 beats per min (bpm) in all HTN patients. In achieving this, higher propensity for various adverse effects including bradycardia with older BB deters their use. Newer BBs like nebivolol must be preferred as they reduce HR as well as both central and peripheral pressures. Further, target HR can be lowered to <65 bpm in patients with coexisting coronary artery disease [24]. In an analysis from SHIFT trial in patients with systolic heart failure, sinus rhythm, and HR≥70 bpm on background of BB treatment, ivabradine addition was associated with significant reduction of composite primary endpoint (CV death or HF hospitalization) and HF hospitalization was reduced significantly in subgroups of no BB (p=0.012) and in all patients <50% of target BB dose. HTN was accompaniment in nearly 2/3^rd^ of the patient population in each subgroup. It was concluded from these results that magnitude of HR reduction with two drugs combined determines the outcomes rather than background BB therapy [25]. In patients with stable CAD receiving stable background *β*-blocker therapy with HR > 70 bpm, addition of ivabradine lowered HR by 15 bpm (from the baseline of 73.8±3.7 bpm) with no change in central BP but was associated with increase in left-ventricular ejection fraction, diastolic perfusion time, and improved myocardial perfusion index. Nearly 83% of the patients had associated HTN. These proofs indicate that, in HTN with comorbidity having HR > 70 bpm despite BB treatment, addition of ivabradine can provide benefits in HF and stable CAD [26].

*Expert Opinion.* In patients with HTN, the target HR remains unclear. Generally, HR<70 bpm on BB treatment is advised which may further be lowered in patients with comorbidities like HF and CAD. Reducing resting HR by at-least 10 bpm from baseline may provide benefits which need to be confirmed in prospective studies.

### 5.2. General Approach to Treatment of HR in HTN

Treatment of an elevated HR in HTN is a subject of debate and there is no clear evidence for optimal approach in management of elevated HR in HTN. The consensus statement endorsed from European Society of Hypertension suggested the following approaches in patients of HTN with high resting HR [6].

- There should be self-measurement of HR in patients who can manage at home
- In patients with high HR in clinic, ambulatory HR measurement should be considered
- Look for secondary causes of increased HR
- There should be emphasis on improvement of lifestyle: increase physical activity, stop smoking, avoid alcohol, and reduce heavy coffee intake
- There should be dietary modification for weight control
- Selective beta-1 blockers have to be considered in symptomatic patients

This consensus document further identifies that in there is no clear evidence to recommend any specific treatment for elevated HR with in HTN that is beneficial and can alter the outcomes. However, in symptomatic tachycardia cases, a selective beta-1 blocker would be safe to use for reducing the HR [6].

### 5.3. Possible Benefits with HR Reduction in HTN

Since publication of ESC consensus document in 2016, we searched for any further evidence studying management of HR in HTN. Relevant literature is discussed in brief here. A recent metanalysis by Xie et al. reported atenolol to be more beneficial than angiotensin converting enzyme inhibitors (ACEIs) in terms of reducing peripheral diastolic pressure and HR in patients of HTN within first 3-months of treatment [27]. Another metanalysis by Nogueira-Silva et al. reported BP reduction by 10/8 mmHg, pulse pressure by 2 mmHg, and HR by 11 bpm with use of BB in HTN [28]. Further, in an interesting analysis from SIMPLICITY registry, Bohm et al. observed that renal denervation in uncontrolled HTN was associated with reduction HR at 12 months of follow-up. The reduction in HR correlated with tertiles of HR at baseline with better reduction in upper tertile of HR (>74 bpm at baseline). They suggested that HR reduction can be a target for renal denervation especially for higher HR at baseline [29]. However, this evidence still did not answer the question whether reducing HR in HTN improves the outcomes in HTN. A possible link to understand effect of lowering HR in HTN in target organ damage has been suggested by a study by Jozwiak et al., where they determined effect of ivabradine on chronic HTN. Administration of ivabradine resulted in 25% reduction in HR from baseline of 86±5 bpm. There was significant improvement in left ventricular twist and untwist which occur in systole and diastole, respectively. This suggests its beneficial effect on LVH in patients of HTN [30].

*Expert Opinion.* Adopt healthy lifestyle approaches to keep check on BP and HR. Use selective beta-1 blocker in symptomatic cases with elevated HR beyond 80-85 mmHg. BBs are expected to lower HR by nearly 10 bpm. It remains unclear whether reducing HR in HTN without comorbidities alters the CV and mortality outcomes.

## 6. Future Research

There is need to generate more data for HR in HTN. We identify the following areas where new research should be focussed to get answers to unanswered questions in Indian setting.

- Deciding on methods of HR assessment at home and in clinic
- Defining a threshold HR for consideration of its treatment
- Defining a target HR (e.g., <70 bpm) or target reduction in HR (e.g., at least 20%) which is safe and effective in HTN
- Assessing effect of lifestyle interventions on HR in uncomplicated HTN
- Assessing CV and mortality outcomes with HR lowering agents (e.g., BB)
- Understanding role of pure HR reducing agents like ivabradine in HTN

## 7. Conclusion

Resting HR is one of the factors found to be associated with incident HTN. Elevated HR is a risk factor and not just a risk indicator. Heart rate in HTN tends to be elevated which has been identified as an independent predictor for adverse cardiovascular and mortality outcomes. It can aid in determining the prognosis of patients with HTN. There remains much uncertainty about optimal approaches to reduce HR in HTN and whether reducing HR improves outcomes in uncomplicated HTN. Use of selective beta-1 blockers is advised in symptomatic cases with elevated HR. There is need of future research to understand the role of HR in HTN and to steer a universal approach in its management. At present, it would suffice to be aware that high heart rate is detrimental in normotensive individuals as well as in hypertensive patients, and, in situations with inappropriately high heart rates, reducing the rate would be justified.

## References

[1] O'Donnell C. J., Elosua R. (2008). Cardiovascular risk factors. insights from Framingham heart study. *Revista Española de Cardiología*.

[2] Wang W., Lee E. T., Fabsitz R. R. (2006). A longitudinal study of hypertension risk factors and their relation to cardiovascular disease: The strong heart study. *Hypertension*.

[3] Miyai N., Arita M., Miyashita K., Morioka I., Shiraishi T., Nishio I. (2002). Blood pressure response to heart rate during exercise test and risk of future hypertension. *Hypertension*.

[4] Palatini P., Dorigatti F., Zaetta V. (2006). Heart rate as a predictor of development of sustained hypertension in subjects screened for stage 1 hypertension: The HARVEST Study. *Journal of Hypertension*.

[5] Palatini P. (2011). Role of elevated heart rate in the development of cardiovascular disease in hypertension. *Hypertension*.

[6] Palatini P., Rosei E. A., Casiglia E. (2016). Management of the hypertensive patient with elevated heart rate: Statement of the Second Consensus Conference endorsed by the European Society of Hypertension. *Journal of Hypertension*.

[7] Reule S., Drawz P. E. (2012). Heart rate and blood pressure: Any possible implications for management of hypertension. *Current Hypertension Reports*.

[8] Roman M. J., Devereux R. B., Kizer J. R. (2007). Central pressure more strongly relates to vascular disease and outcome than does brachial pressure: the strong heart study. *Hypertension*.

[9] Williams B., Lacy P. S., Thom S. M. (2006). Differential impact of blood pressure-lowering drugs on central aortic pressure and clinical outcomes: principal results of the Conduit Artery Function Evaluation (CAFE) study. *Circulation*.

[10] Williams B., Lacy P. S. (2009). Impact of Heart Rate on Central Aortic Pressures and Hemodynamics. Analysis From the CAFE (Conduit Artery Function Evaluation) Study: CAFE-Heart Rate. *Journal of the American College of Cardiology*.

[11] Bangalore S., Sawhney S., Messerli F. H. (2008). Relation of Beta-Blocker-Induced Heart Rate Lowering and Cardioprotection in Hypertension. *Journal of the American College of Cardiology*.

[12] Phillips R. A. (2009). Is the Relationship Between Beta-Blocker-Induced Heart Rate Lowering and Cardiovascular Outcomes the Result of Confounding by Indication?. *Journal of the American College of Cardiology*.

[13] Rao D., Balagopalan J. P., Sharma A., Chauhan V. C., Jhala D. (2015). BEAT survey: A cross-sectional study of resting heart rate in young (18-55 year) hypertensive patients. *Journal of the Association of Physicians of India*.

[14] Wang A., Liu X., Guo X. (2014). Resting heart rate and risk of hypertension: Results of the Kailuan cohort study. *Journal of Hypertension*.

[15] Yang H. I., Kim H. C., Jeon J. Y. (2016). The association of resting heart rate with diabetes, hypertension, and metabolic syndrome in the Korean adult population: The fifth Korea National Health and Nutrition Examination Survey. *Clinica Chimica Acta*.

[16] Singh J. P., Larson M. G., Tsuji H., Evans J. C., O'Donnell C. J., Levy D. (1998). Reduced heart rate variability and new-onset hypertension: Insights into pathogenesis of hypertension: The Framingham Heart Study. *Hypertension*.

[17] Levy R. L., White P. D., Stroud W. D., Hillman C. C. (1945). Transient tachycardia: Prognostic significance alone and in association with transient hypertension. *Journal of the American Medical Association*.

[18] Gillman M. W., Kannel W. B., Belanger A. (1993). Influence of heart rate on mortality among persons with hypertension: the Framingham study. *American Heart Journal*.

[19] Julius S., Palatini P., Kjeldsen S. E. (2012). Usefulness of heart rate to predict cardiac events in treated patients with high-risk systemic hypertension. *American Journal of Cardiology*.

[20] Okin P. M., Kjeldsen S. E., Julius S. (2010). All-cause and cardiovascular mortality in relation to changing heart rate during treatment of hypertensive patients with electrocardiographic left ventricular hypertrophy. *European Heart Journal*.

[21] Okin P. M., Kjeldsen S. E., Julius S. (2012). Effect of changing heart rate during treatment of hypertension on incidence of heart failure. *American Journal of Cardiology*.

[22] Kolloch R., Legler U. F., Champion A. (2008). Impact of resting heart rate on outcomes in hypertensive patients with coronary artery disease: Findings from the INternational VErapamil-SR/ trandolapril STudy (INVEST). *European Heart Journal*.

[23] Hozawa A., Inoue R., Ohkubo T. (2008). Predictive value of ambulatory heart rate in the Japanese general population: The Ohasama study. *Journal of Hypertension*.

[24] Tomlinson B., Dalal J. J., Huang J. (2011). The role of *β*-blockers in the management of hypertension: an asian perspective. *Current Medical Research and Opinion*.

[25] Swedberg K., Komajda M., Böhm M. (2012). Effects on outcomes of heart rate reduction by ivabradine in patients with congestive heart failure: Is there an influence of beta-blocker dose?: Findings from the SHIFT (Systolic Heart failure treatment with the I f inhibitor ivabradine Trial) study. *Journal of the American College of Cardiology*.

[26] Dillinger J.-G., Maher V., Vitale C. (2015). Impact of ivabradine on central aortic blood pressure and myocardial perfusion in patients with stable coronary artery disease. *Hypertension*.

[27] Xie H., Luo G., Zheng Y., Peng F., Xie L. (2017). A meta-analytical comparison of atenolol with angiotensin-converting enzyme inhibitors on arterial stiffness, peripheral blood pressure and heart rate in hypertensive patients. *Clinical and Experimental Hypertension*.

[28] Nogueira‐Silva L., Marques P. S., Lima M. J. (2017). Cochrane corner: antihypertensive efficacy of beta‐1 selective beta blockers for primary hypertension. *Cardiologia*.

[29] Böhm M., Ukena C., Ewen S. (2016). Renal denervation reduces office and ambulatory heart rate in patients with uncontrolled hypertension: 12-month outcomes fromthe global SYMPLICITY registry. *Journal of Hypertension*.

[30] Jozwiak M., Melka J., Rienzo M. (2018). Ivabradine improves left ventricular twist and untwist during chronic hypertension. *International Journal of Cardiology*.

